# Tricyclic Antidepressant Amitriptyline Indirectly Increases the Proliferation of Adult Dentate Gyrus-Derived Neural Precursors: An Involvement of Astrocytes

**DOI:** 10.1371/journal.pone.0079371

**Published:** 2013-11-18

**Authors:** Shuken Boku, Kazue Hisaoka-Nakashima, Shin Nakagawa, Akiko Kato, Naoto Kajitani, Takeshi Inoue, Ichiro Kusumi, Minoru Takebayashi

**Affiliations:** 1 Department of Psychiatry and Behavioral Sciences, Albert Einstein College of Medicine, Bronx, New York, United States of America; 2 Department of Psychiatry, Hokkaido University Graduate School of Medicine, Sapporo, Japan; 3 Department of Pharmacology, Hiroshima University Graduate School of Biomedical Sciences, Hiroshima, Japan; 4 Division of Psychiatry and Neuroscience, Institute for Clinical Research, NHO Kure Medical Center and Chugoku Cancer Center, Kure, Japan; RIKEN Brain Science Institution, Japan

## Abstract

Antidepressants increase the proliferation of neural precursors in adult dentate gyrus (DG), which is considered to be involved in the therapeutic action of antidepressants. However, the mechanism underlying it remains unclear. By using cultured adult rat DG-derived neural precursors (ADP), we have already shown that antidepressants have no direct effects on ADP. Therefore, antidepressants may increase the proliferation of neural precursors in adult DG via unknown indirect mechanism. We have also shown that amitriptyline (AMI), a tricyclic antidepressant, induces the expressions of GDNF, BDNF, FGF2 and VEGF, common neurogenic factors, in primary cultured astrocytes (PCA). These suggest that AMI-induced factors in astrocytes may increase the proliferation of neural precursors in adult DG. To test this hypothesis, we examined the effects of AMI-induced factors and conditioned medium (CM) from PCA treated with AMI on ADP proliferation. The effects of CM and factors on ADP proliferation were examined with BrdU immunocytochemistry. AMI had no effect on ADP proliferation, but AMI-treated CM increased it. The receptors of GDNF, BDNF and FGF2, but not VEGF, were expressed in ADP. FGF2 significantly increased ADP proliferation, but not BDNF and GDNF. In addition, both of a specific inhibitor of FGF receptors and anti-FGF2 antibody significantly counteracted the increasing effect of CM on ADP proliferation. In addition, FGF2 in brain is mainly derived from astrocytes that are key components of the neurogenic niches in adult DG. These suggest that AMI may increase ADP proliferation indirectly via PCA and that FGF2 may a potential candidate to mediate such an indirect effect of AMI on ADP proliferation via astrocytes.

## Introduction

Although many antidepressants are currently available, up to 30% of patients with major depression are still refractory to them [1], [2], [3] and the lifetime prevalence of major depression remains 16.2% in the USA [4]. Therefore, the development of new antidepressants, whose action mechanism is different from existing antidepressants, is ardently desired.

It has been well established that neurogenesis occurs in the dentate gyrus (DG) of adult hippocampus [5], [6]. Neurogenesis contains the proliferation of neural precursors, the differentiation of neural precursors into neurons and the survival of neural precursors and newly born neurons. In these three phenomena of neurogenesis, the proliferation of neural precursors in adult DG is increased by chronic treatments with various classes of antidepressants [7], [8], [9]. In addition, the disruption of the proliferation of neural precursors in adult DG counteracts the behavioral effects of antidepressants [8], [9]. These suggest that the increasing effects of antidepressants on the proliferation of neural precursors in adult DG may be involved in the action mechanism of antidepressants and that increasing the proliferation of neural precursors in adult DG may be beneficial to the treatment of depression. However, it remains unclear how antidepressants increase the proliferation of neural precursors in adult DG.

We have already established the culture system of adult rat DG-derived neural precursors (ADP) [10]. Using ADP, we have already shown that four common mood stabilizers such as lithium, valproate, carbamazepine and lamotrigine, have varied direct effects on ADP proliferation, differentiation and survival [11]. In contrast to mood stabilizers, antidepressants had no direct effect on ADP proliferation, differentiation and survival ([12] and our unpublished data). These suggest that unknown indirect mechanism may mediate the increasing effects of antidepressants on the proliferation of neural precursors in adult DG.

As the candidates of such indirect pathways, two distinct pathways can be considered; neuron-dependent pathway and astrocyte-dependent pathway. We have already shown that noradrenaline (NA) directly increases ADP proliferation through β2-adrenergic receptor [12]. In addition, noradrenergic neurons project from locus coeruleus nucleus to DG [13], [14]. These suggest that antidepressants may increase the proliferation of neural precursors in adult DG through inhibiting NA transporter in noradrenergic neurons. On the other hand, we have recently shown that antidepressants, including tricyclic antidepressant amitriptyline (AMI), induce the expression and secretion of brain-derived neurotrophic factor (BDNF), fibroblast growth factor 2 (FGF2), glial cell-derived neurotrophic factor (GDNF) and vascular endothelial growth factor (VEGF), all of which are known to increase the proliferation of neural precursors in adult DG [15], [16], [17], [18], from primary cultured astrocytes (PCA) [19]. In addition, astrocytes are key components of the neurogenic niches in adult DG [20] and astrocytes derived from adult hippocampus increase the proliferation of adult hippocampal neural precursors in co-culture [21]. These studies suggest that antidepressants may increase the proliferation of neural precursors in adult DG via inducing the expression and secretion of neurotrophic/growth factors from astrocytes. However, it remains unclear whether astrocytes are necessary for the increasing effects of antidepressants on the proliferation of neural precursors in adult DG.

To elucidate the involvement of astrocytes in the effect of antidepressants on the proliferation of neural precursors in adult DG, first we examined the effects of conditioned medium (CM) from AMI-treated PCA on ADP proliferation and showed that CM from AMI-treated PCA increases ADP proliferation. Following it, we examined which neurotrophic/growth factors induced by AMI from PCA are involved in the increasing effect of CM on ADP proliferation.

## Materials and Methods

### Ethical Statement

This study was carried out in strict accordance with the Guidelines for Animal experiments in Hokkaido University and Hiroshima University. The protocols were approved by the Animal Care and Use Committees of Hokkaido University and Hiroshima University. All surgery was performed under sodium pentobarbital anesthesia, and all efforts were made to minimize suffering.

### Isolation and Culture of ADP

ADP were isolated from DG of adult male Sprague-Dawley rats (8 weeks old) as described previously [10]. In brief, ADP were maintained with Neurobasal (Invitrogen, Carlsbad, CA)/B27 supplement (Invitrogen)/1 mM L-glutamine (Invitrogen)/20 ng/ml recombinant basic FGF, identical to FGF2 (Invitrogen) at 37°C on laminin (Invitrogen)-ornithin (Sigma, St. Louis, MO) coated dishes and fed with new medium every two or three days by replacing 50% of the medium.

### Isolation and Culture of PCA and Preparation of CM

PCA were prepared from hippocampus of 1-day-old neonatal Wistar rats. The isolated hippocampus was minced, and then incubated with trypsin (Invitrogen)and DNase (Roche, Indianapolis, IN).Dissociated cells were suspended in Dulbeccco’s Modified Eagle’s Medium (DMEM, Nissui Pharmaceutical, Tokyo, Japan) supplemented with 10% fetal calf serum (Biological Industries, Beit-Haemek, Israel)and penicillin/streptomycin (Nacalai tesque, Kyoto, Japan). Thereafter, cell suspensions were plated in 75 cm^2^ tissue culture flasks (8–15×10^6^ cells/flask) precoated with poly-L-lysine(Sigma).Mixed cells were maintained in a 10% CO_2_ incubator at 37°C. After 8–12 days, cells were separated from neurons and microglia by shaking on a rotary shaker at 100 rpm for 15 h. Adherent cells were trypsinized and plated into 75 cm^2^ flasks. After the cells reached confluence (10 days), the confluent cells were shaken by hand for 10 min. Adherent cells were trypsinized and plated dishes. By this method, >95% of the cells expressed glial fibrillary acidic protein (GFAP), the marker of astrocytes. Medium was changed into Neurobasal/B27/L-glutamate 3 hours before AMI treatment. Then, AMI was added into medium at 0, 25 and 50 *μ*M. These concentrations of AMI were based on our previous study [19]. Medium was collected 48 hours after AMI treatment and used as conditioned medium (CM) for following assays.

### Cell Counting with BrdU Immunocytochemistry

1×10^4^ cells/well were put in laminin-ornithin coated Lab-TekII 8-chamber slides (Nalge Nunc International, Naperville, IL) with FGF2-free medium containing 0.5% FBS (Invitrogen). 24 hours after cell seeding, cells were treated in CM or each drug/neurotrophic factor/antibody. AMI, BDNF and GDNF were purchased from Sigma. SU5402 was purchased from Merck (Darmstadt, Germany). Anti-FGF2 goat polyclonal antibody was purchased from R&D systems (Minneapolis, MN; AB233NA). Normal goat polyclonal IgG antibody (R&D systems, AB108C) was used as a negative control of anti-FGF2 antibody. After 24 hours, bromodeoxyuridine (BrdU, Sigma) was added into medium at 10 nM. After 24 hours, cells were fixed 4% paraformaldehyde for 15 min. Permeabilization was performed as follows; cells were immersed with 50% formamide in 2 X SSC (0.3 M NaCl, 0.03 M sodium citrate) at 65°C for 2 hours, incubated in 2 N HCl for 30 min at room temperature and rinsed in 0.1 M borate buffer for 10 min. Subsequently, cells were incubated in PBS containing 3% goat serum(Vector Laboratories, Burlingame, CA)for 20 min, and then with anti-BrdU mouse monoclonal antibody (1∶50; BD Biosciences, Camarillo, CA, 347580) containing 3% goat serum at 4°C overnight, and incubated in PBS containing Cy3-conjugated goat anti-mouse IgG secondary antibody(1∶100; Jackson Immuno Reserch; 115-165-003) for 1 hour at RT. Samples were coverslipped with Vectashield containing DAPI (Vector Laboratories). Fluorescent signals were detected using IX-71 fluorescent microscope system (Olympus, Tokyo, Japan). The numbers of signals of BrdU and DAPI were counted in randomly selected 4 fields/well. Then, the ratio of BrdU -derived signals/DAPI signals was calculated averaged for each culture. The quantification of data was conducted in a blinded manner. The data was collected from 4 independent cultures. Statistical analysis was performed by one-way ANOVA and Dunnet’s or Bonfferoni’s post hoc test. Significance was defined as *p*<0.05. Data are expressed as the means ± SEM.

### RNA Extraction and RT-PCR Analysis

1×10^5^ cells/well were seeded on laminin-ornithin coated 6-well plates. After overnight incubation, total RNA was extracted from cells with RNeasy extraction kit (Qiagen, Hilden, Germany). Total RNA was converted to cDNA with Quantitect Reverse Transcription kit (Qiagen). PCR was performed with AmpliTaq Gold 360 Master Mix (Applied Biosystems, Foster, CA). The conditions of PCR were: 95°C for 15 min and followed by 35 cycles of 95°C for 30 sec, 60°C for 30 sec 72°C for 30 sec. The sequences of forward and reverse primers for each molecule are as follows; AGC TGG TCA TCA ATG GGA AA and ATT TGA TGT TAG CGG GAT CG for GAPDH, AAC CAA TCG GGA GCA TCT CT and TTG GAA TGT CTC GCC AAC TT for TrkB, GTG TTT GAT GGT GAG GAT GGT and GAG AGA ACA GGG TTC AAG CTG for GDNFR, CCA ACT TTC AAG CAG TTG GTG and GGC ATG GAC AGG TCC AGA TA for FGFR, GGA GAT TGA AAG AAG GAA CGA G and TGG TAC ATT TCT GGG GTG GT for Flk1, AAG CGA TTC ACC TGG ACT GA and CCG CCT CCT TGC TTT TAC TA for Flt1. The PCR products were electrophoresed with 2% agarose gel. The image of this gel was obtained as a digital file and the intensity of each band was analyzed with Image J (National Institutes of Health, Bethesda, MD).

### Detection of FGF2 in CM with Enzyme-limked Immnosorbent Assay (ELISA)

For the assay of the secretion of FGF2, PCA was cultured at a density of 0.9–1.2×10^6^ cells on 35 mm dish with 1 mL of Neurobasal medium supplemented with B27 supplement and 1 mM L-glutamine. CM was collected 2 days after drug treatment and stored at −80°C until assayed. 18 kDa of released form of FGF2 protein levels in CM were determined using a FGF2 enzyme-linked immunosorbent assay kit (R&D Systems) according to the manufacturer’s instructions. Statistical analysis was performed by one-way ANOVA and Dunnet’s post hoc test. Significance was defined as *p*<0.05. Data are expressed as the means ± SEM.

## Results

### Effects of AMI and CM on ADP Proliferation

**Figure 1:**
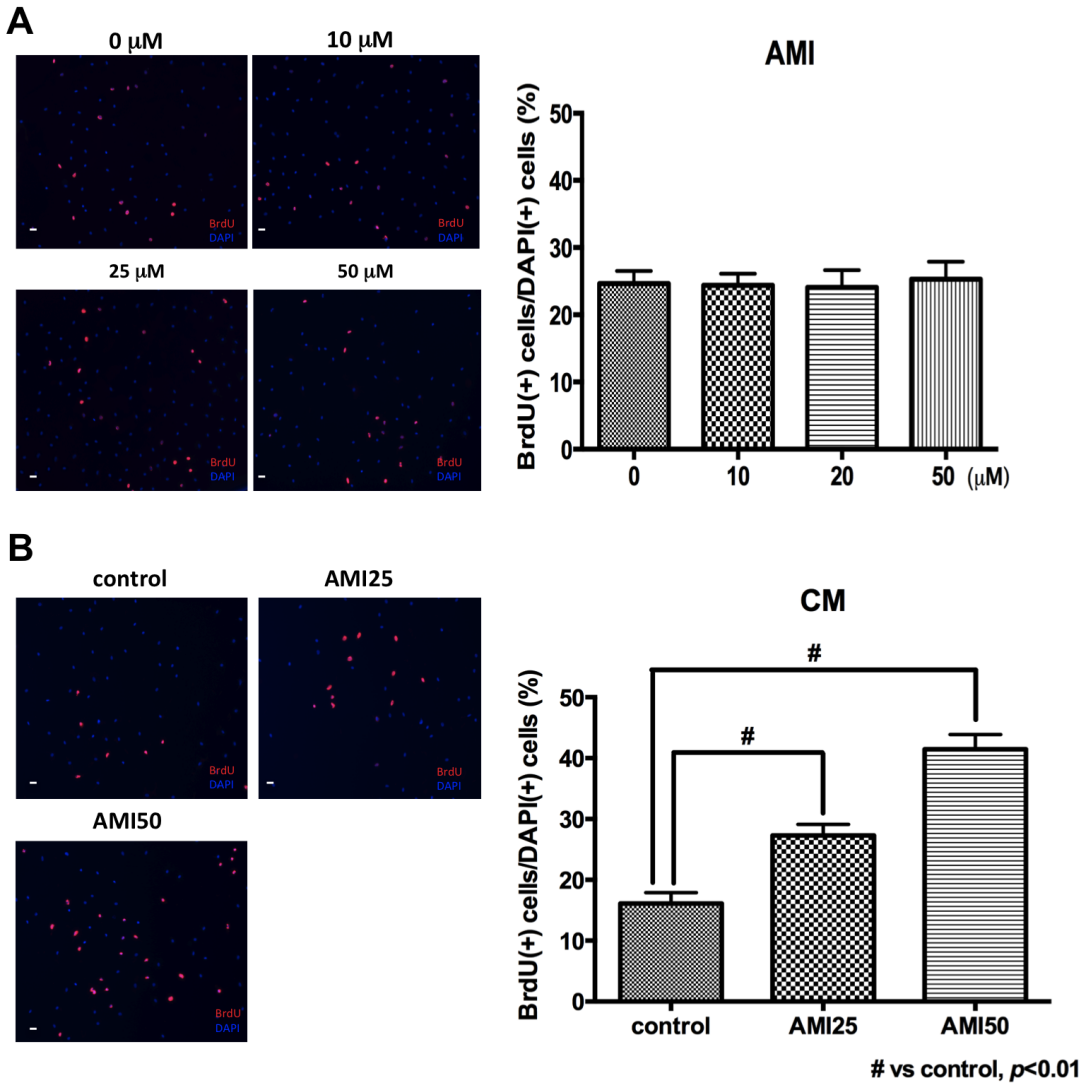
Effects of AMI and CM in ADP proliferation. A. Effect of AMI on ADP proliferation AMI had no effect on ADP proliferation. BrdU was added into medium 1 day after AMI treatment. BrdU immunocytochemistry was perfomed 24 hours after BrdU addition. Data are shown as the means ± SEM. Statistical analysis was performed using one-way ANOVA and Dunnet’s post hoc test. Significance was defined as *p*<0.05, compared with control. Scale bar = 120 *μ*m. B. Effect of CM on ADP proliferation. CM from AMI-treated PCA increased ADP proliferation. Medium was exchanged to 50% CM-containing medium 1 day after plating cells. BrdU was added into medium 1 day after medium exchange. BrdU immunocytochemistry was perfomed 24 hours after BrdU addition. Contrrol means CM from non AMI-treated PCA. AMI25 means CM from 25 µM AMI-treated PCA and AMI50 means CM from 50 µM AMI-treated PCA. Data are shown as the means ± SEM. Statistical analysis was performed using one-way ANOVA and Dunnet’spost hoc test. Significance was defined as *p*<0.05, compared with control. Scale bar = 120 *μ*m.

First, the direct effect of AMI was examined with BrdU immunocytochemistry. AMI had no effect on ADP proliferation (one-way ANOVA and Dunnet’s post hoc test, F(3,28) = 0.05425, *p* = 0.9838, Figure 1A). Next, the effect of CM from AMI-treated PCA was examined with BrdU immunocytochemistry. CM was mixed with equal volume of culture medium (Neurobasal/B27/L-glutamate) and used for assays. CM significantly increased ADP proliferation in an AMI dose-dependent manner (one-way ANOVA and Dunnet’s post hoc test, F(2,45) = 39.37, *p*<0.01, Figure 1B). These results suggest that a factor, whose secretion from PCA is induced by AMI, but not AMI itself, may increase ADP proliferation.

### Effects of FGF2, BDNF and GDNF in ADP Proliferation

**Figure 2:**
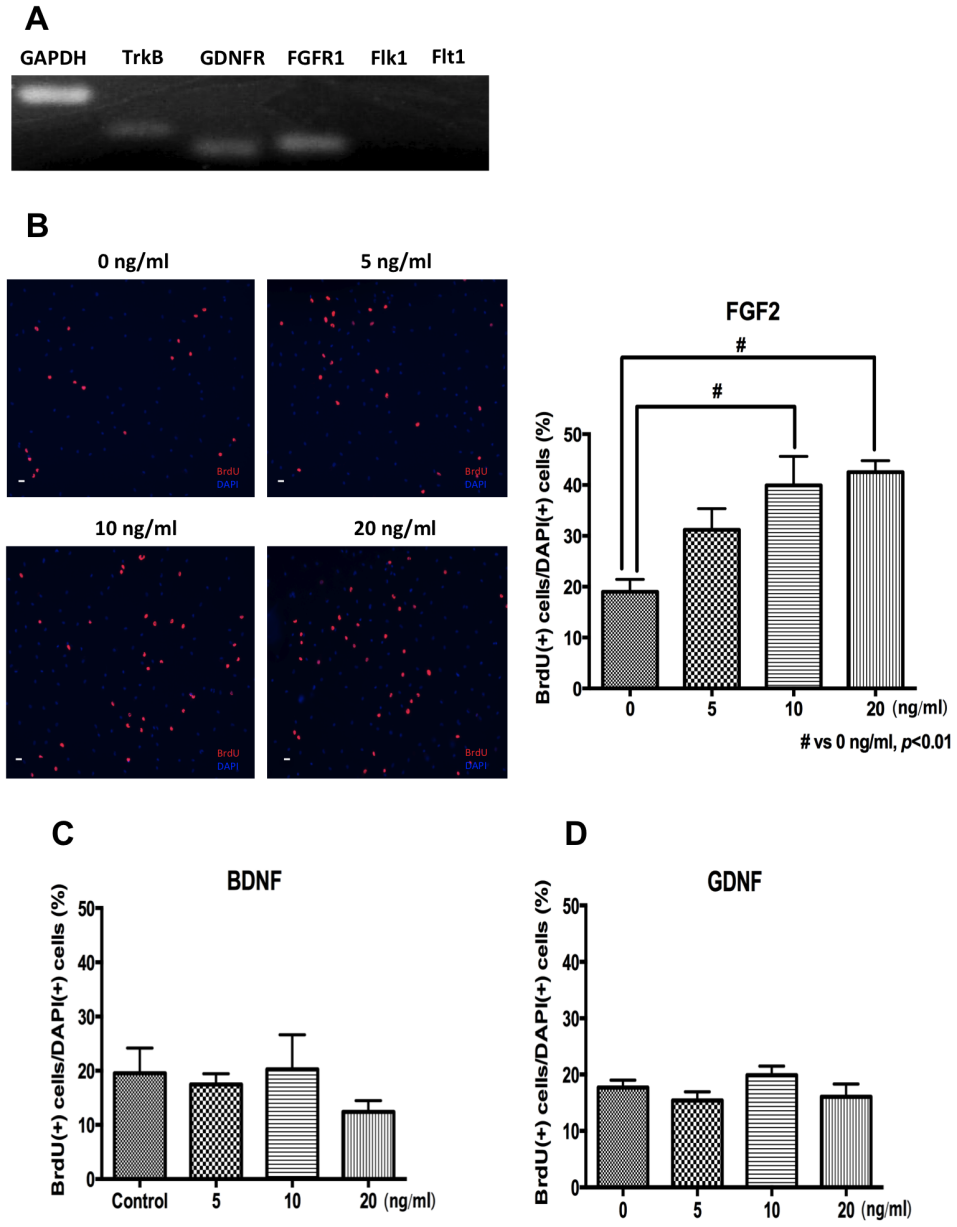
Effects of FGF2, BDNF, GDNF and VEGF in ADP proliferation. A. Detection of mRNA of TrkB, GDNFR, FGFR1, Flk1 and Flt1 mRNA of TrkB, GDNFR and FGFR1 was detected, but mRNA of Flk1 and Flt1, both are receptors of VEGF, were not detected. GAPDH was used as an internal control. B. Effect of FGF2 on ADP proliferation FGF2 increased ADP proliferation in a dose-dependent manner. BrdU was added into medium 1 day after FGF2 treatment. BrdU immunocytochemistry was perfomed 24 hours after BrdU addition. Data are shown as the means ± SEM. Statistical analysis was performed using one-way ANOVA and Dunnet’s post hoc test. Significance was defined as *p*<0.05, compared with 0 ng/ml. Scale bar = 120 *μ*m. C. Effect of BDNF on ADP proliferation BDNF had no effect on ADP proliferation. BrdU was added into medium 1 day after BDNF treatment. BrdU immunocytochemistry was perfomed 24 hours after BrdU addition. Data are shown as the means ± SEM. Statistical analysis was performed using one-way ANOVA and Dunnet’s post hoc test. Significance was defined as *p*<0.05, compared with 0 ng/ml. D. Effect of GDNF on ADP proliferation GDNF had no effect on ADP proliferation. BrdU was added into medium 1 day after GDNF treatment. BrdU immunocytochemistry was perfomed 24 hours after BrdU addition. Data are shown as the means ± SEM. Statistical analysis was performed using one-way ANOVA and Dunnet’s post hoc test. Significance was defined as *p*<0.05, compared with 0 ng/ml.

Our recent study has shown that AMI induces the expression and secretion of FGF2, BDNF, GDNF and VEGF from PCA [19]. It suggests that these four factors are candidates for an inducing factor of ADP proliferation in CM. To confirm it, first, the expressions of receptors for these four factors, FGF receptor 1 (FGFR1) for FGF2, Tyrosine receptor kinase B (TrkB) for BDNF, GDNF receptor 1 (GDNFR1) for GDNF, Fetal liver kinase 1 (Flk1) and Fms-like tyrosine kinase (Flt1) for VEGF, in ADP were examined with RT-PCR. mRNA expression of FGFR1, TrkB and GDNFR1 were detected in ADP, but neither Flk1 nor Flt1 (Figure 2A). Prior to this RT-PCR, the validity of all used primers was confirmed by RT-PCR in which rat brain total RNA (Clontech, Mountain View, CA; data not shown). These results excluded VEGF from a candidate factor of an inducing factor of ADP proliferation in CM. Therefore, the effects of FGF2, BDNF and GDNF on ADP proliferation were examined with BrdU immunocytochemistry. FGF2 significantly increased ADP proliferation in dose-dependent manner up to 10 ng/ml and the effect of FGF2 on ADP proliferation is saturated over 10 ng/ml (one-way ANOVA and Dunnet’s post hoc test, F(3,12) = 7.379, *p*<0.01, Figure 2B). On the other hand, both BDNF and GDNF had no effect on ADP proliferation at 5, 10 and 20 ng/ml (one-way ANOVA and Dunnet’s post hoc test, BDNF: F(3,12) = 0.7215, *p* = 0.5582, Figure 2C; GDNF: F(3,12) = 1.342, *p* = 0.3070, Figure 2D). In addition, BDNF and GDNF had no effect on ADP proliferation at 1, 2, and 50 ng/ml (data not shown). These results suggest that FGF2 may be a potential candidate to mediate the increasing effect of CM on ADP proliferation.

### Effects of AMI on the Secretion of FGF2 from PCA into CM

**Figure 3:**
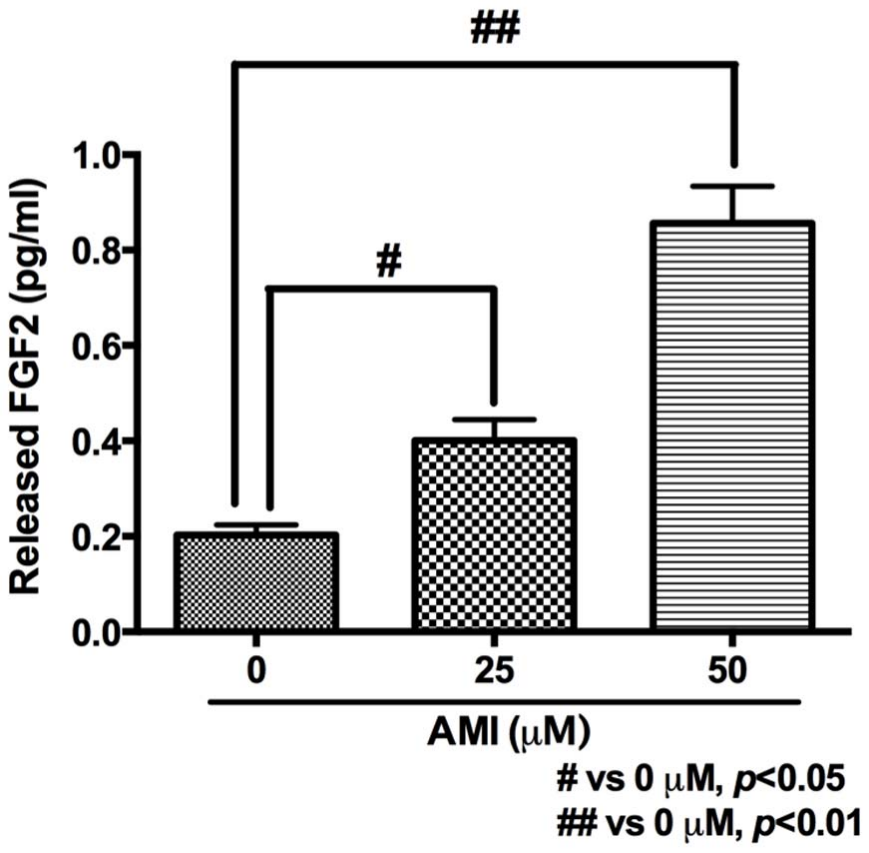
Effects of AMI on the secretion of FGF2 from PCA into CM. AMI induced the secretion of FGF2 from astrocytes into CM in a dose-dependent manner. CM was collected 2 days after AMI treatment. Then, FGF2 in CM was measured with ELISA. Data are shown as the means ± SEM. Statistical analysis was performed using one-way ANOVA and Dunnet’s post hoc test. Significance was defined as *p*<0.05, compared with 0 *μ*M.

To examine whether AMI surely induces the secretion of FGF2 from PCA into CM, the effects of AMI on the concentration of FGF2 in CM was measured with ELISA. AMI significantly increased the concentration of FGF2 in a dose-dependent manner (one-way ANOVA and Dunnet’s post hoc test, F(2,63) = 39.64, *p*<0.01, Figure 3).On the other hand, we have already shown that NA directly increases ADP proliferation [12]. Therefore, to eliminate the possibility that NA is involved in the increasing effect of CM on ADP proliferation, we examined NA concentration in CM from PCA with or without 25 *μ*M AMI for 24 h by HPLC. However, no detectable NA (the detection limit for NA is 0.03 nM) was present in CM [19]. It suggests that AMI induces the secretion of FGF2 from PCA into CM.

### Effects of Anti-FGF2 Antibody and a Specific Inhibitor of FGFR on the Increasing Effect of CM on ADP Proliferation

**Figure 4:**
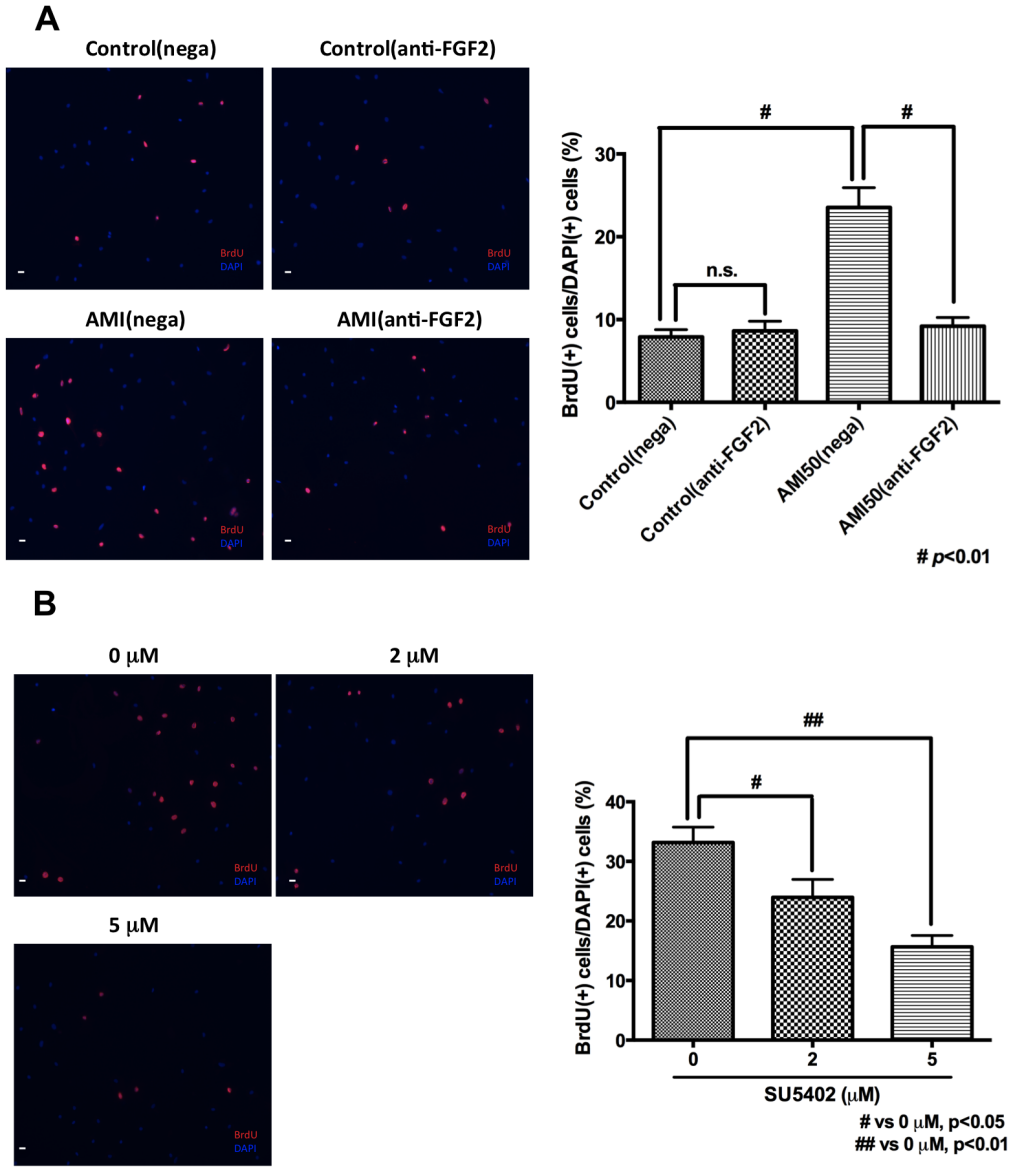
Effects of anti-FGF2 antibody and a specific inhibitor of FGFR on the increasing effect of CM on ADP proliferation. A. Effect of anti-FGF2 antibody on the increasing effect of CM on ADP proliferation Anti-FGF2 antibody counteracted the increasing effect of CM from 50 µM AMI-treated PCA on ADP proliferation. BrdU was added into medium 1 day after antibody treatment. BrdU immunocytochemistry was perfomed 24 hours after BrdU addition. Control means CM from non-treated PCA. Nega means negative control antibody (normal goat IgG). AMI50 means CM from 50 µM AMI-treated PCA. Data are shown as the means ± SEM. Statistical analysis was performed using one-way ANOVA and Bonferroni’s post hoc test. Significance was defined as *p*<0.05. Scale bar = 120 *μ*m. B. Effect of SU5402, a specific inhibitor of FGFR, on ADP proliferation SU5402 counteracted the increasing effects of CM from 50 µM amitriptyline-treated PCA on ADP proliferation in a dose-dependent manner. BrdU was added into medium 1 day after SU5402 treatment. BrdU immunocytochemistry was perfomed 24 hours after BrdU addition. Data are shown as the means ± SEM. Statistical analysis was performed using one-way ANOVA and Dunnet’s post hoc test. Significance was defined as *p*<0.05, compared with 0 nM. Scale bar = 120 *μ*m.

To confirm that FGF2 induced by AMI from PCA into CM is involved in the increasing effect of CM on ADP proliferation, the effects of anti-FGF2 antibody and SU5402, a specific inhibitor of FGFR, on CM from 50 µM AMI-treated PCA were examined with BrdU immunocytochemistry. Both anti-FGF2 antibody and SU5402 had no effect on the number of DAPI (+) cells (data not shown), which means that the ratio of BrdU-derived signals/DAPI-derived signals surely corresponds to cell proliferation. In control, anti-FGF2 antibody had no effect on ADP proliferation. On the other hand, anti-FGF2 antibody significantly counteracted the effect of CM from 50 µM AMI-treated PCA on ADP proliferation (one-way ANOVA and Bonfferoni’s post hoc test, F(3, 28) = 25.00, *p*<0.01, Figure 4A). In addition, SU5402 also significantly counteracted the effect of CM from 50 µM AMI-treated PCA on ADP proliferation (one-way ANOVA and Dunnet’s post hoc test, F(2,33) = 11.93, *p*<0.01, Figure 4B). These results suggest that FGF2 may be a potential candidate to mediate the increasing effect of CM on ADP proliferation.

## Discussion

Here we have shown that CM from AMI-treated PCA, but not AMI itself, increases ADP proliferation. In addition, FGF2 increases ADP proliferation and the secretion of FGF2 from PCA into CM is increased by AMI. On the other hand, BDNF, GDNF and VEGF, whose expressions in PCA are also induced by AMI [19], have no direct effect on ADP proliferation. Moreover, both anti-FGF2 antibody and FGFR inhibitor counteract the increasing effect of CM on ADP proliferation. These results suggest that AMI may indirectly increases the proliferation of neural precursors in adult DG via astrocytes and FGF2 may be a potential candidate to mediate such an indirect effect of AMI on ADP proliferation via astrocytes.

FGF2 is well known to increase the proliferation of neural precursors in adult DG [15]. Astrocytes are involved in neurogenesis in adult DG [20], [21]. In addition, astrocytes are main supply sources of FGF2 [22]. However, there is no past study to show that antidepressants indirectly increase the proliferation of neural precursors in adult DG. Therefore, our present study is the first study to show that antidepressants increase the proliferation of neural precursors in adult DG possibly via inducing FGF2 secretion from astrocytes. On the other hand, we have shown that NA directly increases ADP proliferation through *β*2 adrenergic receptor [12]. Activation of *β*2 adrenergic receptor increases cellular level of cyclic adenosine 3,5–monophosphate (cAMP) [23]. Antidepressants-induced cellular level of cAMP is involved in the proliferation of neural precursors in adult DG [24]. Most of antidepressants including AMI are well known to inhibit NA transporter in neurons and to increase NA levels in the extracellular space. They suggest that antidepressants may increase the proliferation of neural precursors through neurons. Our present study does not exclude this possibility. In addition, our present study was performed *in vitro* and it remains unclear whether antidepressants surely increase the proliferation of neural precursors in *in vivo* adult DG via inducing FGF2 secretion from astrocytes. To examine the possibility that astrocytes and/or neurons mediate the increasing effects of antidepressants on the proliferation of neural precursors in adult DG, selective ablation of astrocytes and neurons in adult DG by some sort of genetic methods may be needed and we will do it in the near future.

Our present study suggests that astrocytes may play a role in the action mechanisms of antidepressants via increasing the proliferation of neural precursors in adult DG and that FGF2 induced by antidepressants from astrocytes may be a potential candidate to mediate it. Past studies have also suggested astrocytes may be involved in the effects of antidepressants in rodents [25], [26], [27], [28]. Therefore, to elucidate how antidepressants induce the secretion of FGF2 from astrocytes may lead to the further understanding the action mechanisms of antidepressants and the development of a new therapeutic target of major depression.

Our previous study has reported that the increasing effect of AMI on FGF2 expression is monoamine-independent and *de novo* protein synthesis-dependent [19]. We also have previously reported that AMI activates FGFR through matrix metalloproteinase (MMP)-dependent shedding of FGFs in a monoamine-dependent manner in C6 cells, a model of astrocytes [29]. Therefore, we are currently performing further studies whether the activation of FGFR through MMP might be involved in AMI-induced FGF2 expression in astrocytes. Clarifying monoamine-independent novel targets of antidepressants in astrocytes might contribute to the development of more efficient treatments for major depression.

Recombinant FGF2, produced in E.coli, significantly increases ADP proliferation at 10 ng/ml (Figure 2B). On the other hand, the concentration of FGF2 in AMI-treated CM is 0.4–0.8 pg/ml (Figure 3). Thus, there is significant discrepancy between effective concentrations of recombinant FGF2 and PCA-derived FGF2 concentrations. At first glance, it seems like a contradiction. However, we can consider this discrepancy may be reasonable, not strange, by the following reasons. It is well known that recombinant proteins produced in E.coli generally lack post-translational modification. On the other hand, post-translational modification of heparin sulfate proteoglycan significantly increases the binding activity of FGF2 to FGFR [30]. In addition, cystatin C is required for FGF2-denpendent proliferation of neural precursor cells in adult DG [31]. Moreover, cystatin C is produced in astrocytes [32], [33]. These suggest that the binding activity of FGF2 from PCA may be much higher than that of recombinant FGF2 and/or that co-secreted cystatin C from PCA may significantly enhances the binding activity of FGF2 in CM from AMI-treated PCA.

Past studies have shown that FGF2 is involved in cell survival [34]. On the other hand, both anti-FGF antibody and SU5402, a specific inhibitor of FGFR, seemed to have no effect on the number of DAPI (+) cells to the extend we examined. It suggests that both anti-FGF2 antibody and SU5402 may not decrease cell number under our experimental condition. We think that FGF2 may be involved in ADP survival and that 0.5% FBS contained in medium and some growth factors/neurotrophic factors contained in CM may also be involved in ADP survival. Therefore, 0.5% FBS in medium and some growth factors/neurotrophic factors contained in CM might compensate the effect of FGF2 on ADP survival and then both anti-FGF2 antibody and SU5402 might not decrease cell number under our experimental condition.

Only AMI was used as an antidepressant in our present study because all of different classes of antidepressants, tricyclic antidepressants, selective serotonin reuptake inhibitors, serotonin noradrenaline reuptake inhibitors, similarly increase FGF2 expression in PCA [19]. We have shown that none of various classes of antidepressants have any direct effect on ADP proliferation ([12] and our unpublished data). In addition, previous studies have reported that various classes of antidepressants increases mRNA expression of neurotrophic/growth factors in rodent cortical astrocyte cultures [35], [36] and that the reduction of GFAP, a common marker of astrocytes, induced by chronic unpredictable stress in the hippocampus is reversed by clomipramine, another tricyclic antidepressant, accompanied by the improvement of depressive-like behavior [37]. These suggest that the indirect increasing effect of AMI on ADP proliferation through astrocytes may be common among antidepressants.

A past study showed that antidepressants did not increase the proliferation of neural progenitor cells from neonatal rats through astrocytes by using co-culture system [38]. However, Ko et al’s study has a serious problem. 20 ng/ml of FGF2 is added in medium through all experiments in [38]. We also used 20 ng/ml FGF2 to maintenance the culture of ADP, but completely removed FGF2 from medium 24 hours before the treatment of AMI, CM and neurotrophic/growth factors, because our present study suggests that the effect of FGF2 on the proliferation of neural precursor cells is saturated at 10 ng/ml (Figure 2B). These suggest that the presence of 20 ng/ml FGF2 in medium may mask the increasing effect of astrocytes on the proliferation of neural progenitor cells from neonatal rats in [38]. Therefore, Ko et al’sstudy has a serious problem and does not negate our results.

Here we have shown that tricyclic antidepressant AMI may increase the proliferation of neural precursor cells from adult DG via astrocytes and that FGF2 may be a potential mediator of such an indirect effect of AMI. It suggests that FGF2 from astrocytes may be a candidate to play an important role in the action mechanisms of antidepressants through neurogenesis in adult DG. However, in order to make a conclusion, it should be examined whether the recombinant FGF2 and CM have the same effect by analyzing phosphorylation level of FGFR or the activation level of downstream targets of FGFR. Moreover, it remains unclear how antidepressants induce FGF2 secretion from astrocytes. To elucidate it might lead to the discovery of monoamine-independent novel action mechanisms of antidepressants.
